# Factors Associated with Mental Health Problems Among Tuberculosis Patients Attending Tertiary Care Hospitals in the Bangkok Metropolitan Region, Thailand: A Hospital-Based Survey

**DOI:** 10.3390/clinpract15030043

**Published:** 2025-02-22

**Authors:** Kanjana Konsaku, Titaporn Luangwilai, Parichat Ong-Artborirak

**Affiliations:** Department of Research and Medical Innovation, Faculty of Medicine Vajira Hospital, Navamindradhiraj University, 681 Samsen Road, Dusit, Bangkok 10300, Thailandtitaporn.lua@nmu.ac.th (T.L.)

**Keywords:** mental health, psychological distress, depression, tuberculosis, urban

## Abstract

**Background:** Little is known about mental health among tuberculosis (TB) patients in Thailand. This study aimed to identify factors associated with mental health problems in TB patients in urban Thailand. **Methods:** This cross-sectional study collected data from 210 TB patients receiving treatment at two tertiary care hospitals in the Bangkok Metropolitan Region of Thailand using consecutive sampling. The General Health Questionnaire (GHQ-12) and the Patient Health Questionnaire (PHQ-9) were used to assess mental health problems and probable depression, respectively. **Results:** Among TB patients, 34.3% had mental health problems (95% CI: 27.8–40.8), and 23.8% had depression (95% CI: 18.0–29.6). The final model from logistic regression with forward selection identified factors significantly associated with mental health problems, including high family support (OR = 0.45; 95% CI: 0.24–0.83) and perceived stigma: low (OR = 2.77; 95% CI: 1.16–6.60), moderate (OR = 3.56; 95% CI: 1.66–7.65), and high (OR = 3.56; 95% CI: 1.31–9.67) versus no stigma. Depression was associated with income ≥10,000 baht (OR = 0.43; 95% CI: 0.21–0.87), alcohol consumption (OR = 2.90; 95% CI: 1.27–6.64), and high social support from healthcare providers (OR = 0.44; 95% CI: 0.22–0.87). **Conclusion:** This study highlights the need to integrate mental health services into the TB care program to address the TB challenge in Thailand. Policies such as routine mental health screening and psychological counseling alongside treatment, as well as expanded social support and stigma reduction interventions, should be implemented to reduce the risk of mental health issues, particularly depression, and improve treatment outcomes among Thai TB patients.

## 1. Introduction

Tuberculosis (TB) is a communicable disease caused by the bacterium *Mycobacterium tuberculosis*, posing a significant medical and public health challenge worldwide. In 2023, the World Health Organization reported a global TB incidence rate of 134 per 100,000 population, equivalent to 10.8 million people [1]. In Thailand, an estimated 113,000 people contracted TB in 2023, translating to 157 cases per 100,000 population, with 13,000 TB-related deaths [2]. Although Thailand has a national action plan to combat TB, focusing on prevention, care, treatment, and control measures, the number of newly identified and registered TB cases—both new and relapsed—continues to rise [3]. This increase is particularly evident in Bangkok, which has the highest number of new and relapsed TB cases in the country [4].

A TB diagnosis can cause overwhelming feelings of shock, shame, and anxiety about becoming seriously ill, dying, or infecting loved ones, while also leading to isolation, job loss, and stigma that can impact patients’ self-esteem [5,6]. Consequently, TB can lead to mental health problems, particularly depression, and negatively impact quality of life [7,8,9]. Depression can further undermine health behaviors (e.g., diet, healthcare seeking, and medication adherence), presenting a major barrier to the global elimination of TB [10]. Additionally, depression contributes to negative treatment outcomes, including increased morbidity, mortality, community transmission, and antibiotic resistance [10,11,12]. While mental healthcare is incorporated into the TB care guidelines in Thailand, its implementation remains unclear and lacks concrete actions.

The burden of mental disorders has increased and continues to rise worldwide [13]. Psychological distress among TB patients has been reported in various countries, ranging from 22% to 84%, with depression reported in the range of 9% to 84% [5]. A meta-analysis estimated the prevalence of depression among TB patients at 11% [14]. In low- and middle-income countries (LMICs), depressive episodes are more prevalent in adults with TB compared with those without (23.7% vs. 6.8%), with a strong association observed in the Asian region [15]. In Southeast Asia, the prevalence of depression among TB patients ranges from 7.7% to 39.1% [16,17,18,19,20], with Thailand reporting 7.8% to 20.4% [7,21]. Notably, Thai adults in urban areas, such as Bangkok, have a much higher prevalence compared with those in rural areas [22] due to unique urban stressors and factors including overcrowding, a fast-paced lifestyle, violence, and low social support [22,23]. Additionally, depression in TB patients is associated with various factors, including sociodemographic factors (e.g., sex, age, education, marital status, and income), clinical factors (e.g., retreatment status and duration of illness), and psychosocial factors (e.g., social support and perceived stigma) [24,25,26,27,28,29,30,31,32,33].

Although previous studies in Thailand have explored mental health in TB patients, it has received limited attention, and no studies have identified the factors associated with it. Furthermore, findings from other countries may not be generalizable to the Thai population due to differences in characteristics. Therefore, this study aimed to investigate the factors associated with mental health problems in TB patients in Bangkok and its metropolitan area. A cross-sectional study was conducted to assess a wide range of mental health problems, particularly depression, while considering potential factors based on existing research from multiple countries, including sociodemographic, clinical, and psychosocial variables. The findings can inform the planning of TB patient care, from diagnosis to treatment, to prevent mental health issues and negative treatment outcomes.

## 2. Materials and Methods

The study was conducted in two areas: Bangkok, the capital of Thailand, which has the highest number of TB patients in the country, and Samut Prakan Province, a metropolitan area with a large number of registered TB patients. Two state tertiary care hospitals were purposively selected based on pragmatic considerations, including their feasibility for data collection and catchment populations: Vajira Hospital, the largest operated by the Bangkok Metropolitan Administration, and Samut Prakan Hospital, the main government hospital of the province. The inclusion criteria were as follows: TB patients diagnosed and registered for treatment, aged 20 years and above, residing in Bangkok or its metropolitan area for at least 6 months, able to communicate in Thai, and not undergoing psychiatric treatment according to their self-report.

The sample size was determined using a rule of thumb for regression analysis, Thorndike’s formula (n = 10k + 50, where n is the sample size and k is the number of independent variables) [34]. This study preliminarily investigated a total of 16 factors, resulting in a sample size of 210 participants. Consecutive sampling was used to select participants by promoting the research to TB outpatients receiving treatment at the TB clinics of both hospitals. All eligible TB patients attending clinic services on that day were sequentially invited to participate in the study while waiting for their follow-up appointments with the doctor. The researcher ultimately collected data from 106 patients at Vajira Hospital and 104 patients at Samut Prakan Hospital between March and May 2024.

In general, the hospital’s medical team routinely contacts TB patients to remind them about their follow-up appointments and invites those who miss their scheduled appointments to visit the clinic. This strategy may minimize selection bias by including patients who might otherwise be overlooked, such as those who are irregular in attending follow-up visits. By reaching a broader range of patients, the recruitment process reduces the risk of overrepresenting regular clinic attendees. This approach may result in a more diverse and representative sample.

The study was approved by the Human Research Ethics Committee of the Faculty of Medicine, Vajira Hospital, Navamindradhiraj University (COA No. 050/2567), and by the Human Research Ethics Committee of Samut Prakan Hospital (No. Tq02667). Participants who agreed to take part signed a written informed consent form before the researcher conducted face-to-face interviews to collect data.

The interviewer-administered questionnaire consisted of three parts. Part 1 was a general information questionnaire (16 items) covering personal characteristics and TB-related factors, based on previous research [24,25,26,27,28,29,30,31,32,33]. The items included sex, age, marital status, education, employment status, monthly income, smoking status, alcohol consumption, body mass index (BMI), chronic diseases, patient category, TB type, treatment duration, social support from healthcare providers, social support from family, and perceived stigma. Smoking status was assessed by asking patients whether they were current smokers (no/yes). Current alcohol consumption was defined as drinking monthly, weekly, or daily. BMI was calculated based on self-reported weight and height, while the presence of chronic diseases was based on self-reported diagnoses. Patients were asked to rate their level of social support from healthcare providers (e.g., information on TB prevention and treatment, encouragement and empathy, friendly support from doctors, nurses, or healthcare staff, and material assistance) and from family members (support in TB care), as perceived by themselves as low, moderate, or high. Perceived stigma was assessed using a single question about their perception of whether individuals with TB are stigmatized, devalued, or discriminated against by society, with ratings on four levels: none, low, moderate, and high. Some self-reported data were randomly cross-checked with the hospital’s database to ensure accuracy and reliability.

Part 2, the General Health Questionnaire (GHQ-12), is a 12-item screening tool for mental health and psychological distress [35], which has been translated and validated for Thai people [36]. It uses a 4-level rating scale (bi-modal: 0-0-1-1), with a score range of 0 to 12. In accordance with the cut-off scores used in other countries, a score of 2 or above indicates the presence of mental health problems [36]. The instrument has a Cronbach’s alpha coefficient of 0.86, sensitivity of 0.78, specificity of 0.85, and an area under the curve (AUC) of 0.89 [36]. Part 3, the Patient Health Questionnaire (PHQ-9), is a 9-item screening tool for major depression [37], which has been translated into Thai and its psychometric properties tested [38]. Each item is rated on a 4-level scale (0-3), with a score range of 0-27. Although global standards recommend a cut-off score of 10 points [37], this study used a score of 9 or higher to indicate probable depression, as recommended for use in clinics for the Thai version [38]. The tool has a Cronbach’s alpha coefficient of 0.79, sensitivity of 0.84, specificity of 0.77, and an AUC of 0.89 [38]. The GHQ-12 and PHQ-9 were chosen for this study because they are Thai-validated versions with demonstrated reliability, indicating their suitability for use. The questionnaire took approximately 15–20 min per patient to complete.

All statistical analyses were conducted using SPSS Version 28 (IBM Corp., Armonk, NY, USA). The independent variables and mental health outcomes were characterized using descriptive statistics, including number (n), percentage (%), mean, standard deviation (SD), minimum (Min), and maximum (Max). The Chi-square test was used to assess the proportions of TB patients with mental health problems, including depression, across each independent variable. Binary logistic regression with forward Wald selection was used to identify factors associated with the study outcomes, which were obtained from the Chi-square test with a *p*-value < 0.2. Odds ratios (ORs) and their 95% confidence intervals (95% CIs) for the final model were reported to show the strength of the association. Variance inflation factors (VIF) for each model were tested and showed no evidence of collinearity.

## 3. Results

Among 210 TB patients, 34.3% had mental health problems (95% CI: 27.8–40.8), and 23.8% had probable depression (95% CI: 18.0–29.6), as shown in Table 1. Personal factors and TB-related information for these patients are presented in Table 2. The patients were predominantly male (63.8%), with a mean age of 49.7 years (SD = 17.3). The majority were single (42.4%), and a low percentage reported having no formal education (8.6%). A significant portion were employed (67.6%), had a monthly income of ≥10,000 baht (51.0%), did not smoke (87.6%), and did not consume alcohol (81.9%). The mean BMI was 20.6 kg/m^2^ (SD = 3.8), with most having a normal BMI (41.9%). Chronic diseases were reported by 50.5% of patients, with the most common being diabetes (43.4%), followed by hypertension (41.5%), dyslipidemia (26.4%), and allergies (16.0%).

Regarding TB clinical data, most patients were newly diagnosed with TB (89.0%) and had pulmonary TB (93.3%). The mean duration of TB treatment was 3.77 months (SD = 3.30), with a range from one week to 18 months. Patients reported varying levels of social support from healthcare providers as follows: high (53.8%), moderate (37.1%), and low (9.1%). Similarly, social support from family members was reported as high (61.9%), moderate (24.3%), and low (13.8%). In terms of stigma, 35.2% did not perceive any stigma related to their condition, while 64.8% reported feeling stigma.

The Chi-square test showed significant differences in the proportion of mental health problems across five variables: age, alcohol consumption, social support from healthcare providers, social support from family, and perceived stigma (all *p*-values < 0.05). A significant difference in the proportion of depression was found for three variables: alcohol consumption, social support from healthcare providers, and social support from family (all *p*-values < 0.05) (Table 2).

The logistic regression analysis using the forward method to identify factors associated with mental health problems is presented in Table 3. Statistically significant associations were found for high family support (OR = 0.45; 95% CI: 0.24–0.83) versus low to moderate family support, and perceived stigma: low (OR = 2.77; 95% CI: 1.16–6.60), moderate (OR = 3.56; 95% CI: 1.66–7.65), and high (OR = 3.56; 95% CI: 1.31–9.67) versus no stigma. However, significant variables from the univariable analysis, such as age, alcohol consumption, and social support from healthcare providers, were not included in the final model. Additionally, factors significantly associated with depression included income ≥10,000 baht (OR = 0.43; 95% CI: 0.21–0.87) versus income <10,000 baht, alcohol consumption (OR = 2.90; 95% CI: 1.27–6.64) versus no consumption, and high social support from healthcare providers (OR = 0.44; 95% CI: 0.22–0.87) versus low to moderate support, as shown in Table 4. Monthly income became a significant factor in the final model, whereas social support from family was excluded.

## 4. Discussion

This study found that 34% of TB patients at two tertiary care hospitals in urban areas experienced mental health problems, with 24% showing probable depression. The findings suggest the need for enhanced mental health support for high-risk Thai TB patients, including additional care and surveillance to reduce the potential negative impact on treatment outcomes and improve their quality of life [7,8,9]. According to a mixed-methods systematic review and meta-analysis, psychosocial support interventions, including material support (financial and nutritional) and psychological support (counseling and health education), can improve treatment outcomes among TB patients [39]. In LMICs, implementing person-centered TB care models within routine care platforms is beneficial [5]. These findings emphasize the importance of integrating routine mental health screening into TB care models in Thailand, despite the practical constraints of limited resources. Psychological counseling can be effectively implemented once patients’ mental health is identified and monitored. Short training modules could enhance the feasibility of this approach, as they require minimal additional time from healthcare professionals.

According to existing evidence, TB infection and the stress associated with the disease can disrupt the hypothalamic–pituitary–adrenal (HPA) axis, resulting in altered cortisol levels, as observed in patients with depression [40,41,42]. TB infection can also trigger the release of pro-inflammatory cytokines, such as Interleukin-6 (IL-6) [43], which may contribute to depression [44]. This can further impact health behaviors, such as diet, healthcare seeking, and medication adherence, thereby worsening treatment outcomes [10]. These findings may inform future interventional studies, such as identifying therapeutic targets to reduce TB-related inflammation. Along with addressing the physical aspects, intervention development should also target stressors, including social and emotional factors, to enhance treatment effectiveness. However, this cross-sectional study cannot confirm that mental health problems in TB patients are a direct result of TB infection. This approach does not allow for the examination of changes in mental health status over time. The use of screening tools instead of clinical diagnoses may also lead to overestimation or underestimation. Since this study collected data from patients who had follow-up visits at hospitals, it is possible that the reported prevalence of mental health problems may underestimate the actual situation. TB patients with depression are more likely to be lost to follow-up [12].

Compared with other studies using the GHQ-12, the prevalence in this study is lower than those reported in India (74%) [45] and Nigeria (75%) [46]. When considering other studies that used the PHQ-9 assessment tool, the prevalence of depression in this study is also lower than in Ethiopia (52-54%) [25,26,30], India (58%) [47], Cameroon (61%) [48], and Afghanistan (70%) [49], but higher than in Malaysia (8%) [17], Myanmar (10%) [18], and Nepal (10%) [33]. However, the findings are consistent with a previous study in Thailand (20%) [7], as well as studies from Southeast Asian, including the Philippines (17%) [16], Vietnam (26% for non-MDR) [19], and other countries such as China (18%) [50] and Ethiopia (31%) [31]. In addition to differences in TB patient characteristics and study settings, including cultural and health system variations, these discrepancies may partly result from the use of varying cut-off scores for categorizing and defining depression. For instance, 5 points are used to define mild depression [26,30,47,48,49], while 10 points are used for probable depression [16,17,18,25,31,33,50].

Mental health problems in TB patients were significantly associated with family social support and perceived stigma, while an increased risk of depression was linked to monthly income, alcohol consumption, and social support from healthcare providers. In addition, the age group was also associated with mental health problems in the univariable model. TB patients aged 60 years and older had the lowest risk of mental health problems compared with other age groups. This is similar to a study of adult participants in Australia, which found that older age was associated with a lower risk of psychological distress in the unadjusted analysis [51].

Good social support from family members is associated with a 55% reduced risk of mental health problems. In terms of healthcare providers, high support was identified as a protective factor against depression, lowering the risk by 56%. This is similar to the findings from previous studies, which showed that social support was significantly associated with depression in Ethiopia [25,26,29,31], Afghanistan [49], and Cameroon [27]. Both objective and subjective support were found to be inversely related to the presence of depressive symptoms in Chinese patients with TB [52]. The Thai study found that strong family support, both mentally and financially, helped TB patients cope better with stress, whereas those with weak support experienced suicidal thoughts due to their family’s fear of transmission, and the lack of care or encouragement made them feel lonely and depressed [53]. According to the Thai TB control program guidelines, support from healthcare providers can include TB education, encouragement, companionship, and material assistance [3]. These are based on theoretical evidence of perceived social support, including emotional, informational, and tangible support [54]. In addition, a meta-analysis found that material support is both feasible and effective when combined with other social support interventions for TB patients in LMICs [55]. The findings highlight the importance of both expanded family support and healthcare provider support in safeguarding the mental health of Thai TB patients. This is particularly important in urban settings, where low social support may be more prevalent [23].

Perceived stigma is associated with an elevated risk of mental health problems, particularly at moderate and high levels. This finding is in accordance with research in Ethiopia [56], which found that patients who experienced TB stigma had 1.7 times higher odds of psychological distress than those who did not. A high level of experienced stigma (OR = 2.24) was a significant predictor of psychological distress compared with a low level [57]. Stigmatization can lead to self-isolation both at the workplace and at home, particularly among MDR-TB patients, who are highly stigmatized due to fears of disease transmission to others in the community [6,53]. Patients with TB may also develop negative thought patterns, such as catastrophizing, which can contribute to mental health problems [58]. A Thai survey on stigma and discrimination found that discrimination in the family, workplace, and health service establishments decreased by 8.1%, 6.7%, and 3.3%, respectively, while the self-stigma of TB patients remained unchanged, including negative self-perception, feelings of shame, hiding their condition, and avoiding social interactions [59]. These are based on stigma mechanisms, including experienced, anticipated, and internalized stigma [60,61]. The findings indicate the importance of ongoing measures to reduce TB-related stigma and minimize mental health problems among Thai patients in urban areas, where stressors such as overcrowding may exacerbate stigma. TB-stigma-reduction interventions may primarily target three key populations: individuals with TB (e.g., home visits, TB clubs, and psychosocial support groups), healthcare workers (e.g., training, workshops, and social marketing campaigns), and the public (e.g., educational materials and mass gatherings) [62]. Practical measures, such as community campaigns and peer-led support groups, could be integrated into the Thai TB program to mitigate stigma.

A lower monthly income is linked to a higher risk of depression. This finding is consistent with a study in China, which found that TB patients with higher household monthly income had significantly lower depression scores (3.28 points) [52]. In Ethiopia, lower levels of income were found to increase the risk of depression [30]. Even with free anti-TB drugs, low income can make it hard to cover additional treatment-related costs such as nutrition, transportation, and missed work, leading to lower earnings and psychological distress from the inability to meet the needs of the individual and their household [30,63]. Poverty may also contribute to depression by increasing exposure to violence, social exclusion, discrimination, and abuse, while limiting access to healthcare, education, and essential services [10,64]. Similar to the Thai urban context, financial strain from low income may increase social stigma and make it difficult to access care or treatments, including nutrition and transportation, especially due to overcrowded living conditions.

The odds of depression are 2.9 times higher in TB patients with current alcohol consumption compared with those without. This finding is similar to a study in Nepal [33], which found that TB patients with current alcohol use had PHQ scores 1.73 points higher than those without alcohol use. Data from the Health Survey for England show that alcohol consumption, particularly heavy drinking (abuse or dependence), can contribute to depression [65]. Alcohol consumption can impair physical functioning and hinder emotional coping, while also indirectly leading to social and financial problems, ultimately damaging mental health. However, depression tends to result from drinking in men, whereas in women, depression is often an antecedent to drinking problems [65]. In general, Thai males are more likely to consume alcohol than Thai females. This trend is also reflected in the results of this study, which indicates potential gender differences and a link to depression among TB patients. Additionally, alcohol consumption can increase the chances of relapse, worsen clinical outcomes, and contribute to the development of MDR-TB [66].

Regarding the study’s limitations, a hospital-based cross-sectional study using non-probability sampling may not establish causal relationships between variables and may not be representative of the Thai population of TB patients, limiting its generalizability. Not only may selection bias occur, but also response bias, particularly if participants were uncomfortable reporting mental health concerns. The sample size is relatively small, and some factors were not investigated. The study used a screening tool to assess mental health problems, including depression, rather than clinical diagnoses made by healthcare professionals. As this is a preliminary survey among Thai TB patients in urban areas, a larger sample size, the use of standard tools, and the inclusion of additional factors, such as access to mental health services, are needed for future studies to confirm the findings. Further research should include longitudinal designs to address the current limitations. Qualitative approaches could also provide deeper insights and enrich the understanding of TB-related mental health challenges in the Thai context.

## 5. Conclusions

In this study, mental health problems, including depression, in TB patients were associated with personal factors such as age, income, and alcohol consumption, as well as psychosocial factors such as social support and perceived stigma. These findings suggest the integration of mental health services into the National TB Control Program, with concrete actions for policymakers. The implementation of validated screening tools to identify and monitor mental health at each follow-up visit, coupled with psychological counseling services in TB clinics, should be prioritized to address mental health issues early through collaborative care. These should include short training modules and the establishment of quick referral pathways for on-site TB clinic staff. Additionally, enhanced interventions for social support, especially material support, along with strengthened efforts to reduce stigma and a focus on the delivery of care, should be considered. Training in social support and stigma reduction for both healthcare providers and family members is necessary. For researchers, studying the effects of social support and stigma reduction interventions for TB patients is recommended to improve their mental health and well-being, as well as to prevent negative treatment outcomes.

## Figures and Tables

**Table 1:** Descriptive data on the GHQ-12 and PHQ-9 questionnaires among TB patients (*n* = 210).

Outcome	*n* (%)	Mean (SD)	Min–Max
GHQ-12		1.80 (2.88)	0–12
No mental health problems (0–1 points)	138 (65.7)		
Mental health problems (2–12 points)	72 (34.3)		
PHQ-9		5.43 (4.83)	0–27
No depression (0–8 points)	160 (76.2)		
Probable depression (9–27 points)	50 (23.8)		

**Table 2:** Personal characteristics, clinical factors, and psychosocial aspects of TB patients, categorized by the presence of mental health problems and depression *(n* = 210).

Factors	All [n (%)]	Mental Health Problems			Depression		
		No [n (%)]	Yes [n (%)]	*p*-Value	No [*n* (%)]	Yes [n (%)]	*p*-Value
Sociodemographic factors							
Sex				0.534			0.521
Male	134 (63.8)	86 (64.2)	48 (35.8)		104 (77.6)	30 (22.4)	
Female	76 (36.2)	52 (68.4)	24 (31.6)		56 (73.7)	20 (26.3)	
Age				**0.019**			0.583
<30 years	42 (20.0)	23 (54.8)	19 (45.2)		30 (71.4)	12 (28.6)	
30–44 years	39 (18.6)	24 (61.5)	15 (35.5)		30 (76.9)	9 (23.1)	
45–59 years	59 (28.1)	35 (59.3)	24 (40.7)		43 (72.9)	16 (27.1)	
≥60 years	70 (33.3)	56 (80.0)	14 (20.0)		57 (81.4)	13 (18.6)	
Marital status				0.572			0.417
Married	84 (40.0)	57 (67.9)	27 (32.1)		68 (81.0)	16 (19.0)	
Single	89 (42.4)	55 (61.8)	34 (38.2)		65 (73.0)	24 (27.0)	
Divorced/separated/widowed	37 (17.6)	26 (70.3)	11 (29.7)		27 (73.0)	10 (27.0)	
Education				0.466			0.898
No education/primary school	94 (44.8)	66 (70.2)	28 (29.8)		73 (77.7)	21 (22.3)	
Secondary school/diploma	79 (37.6)	49 (62.0)	30 (38.0)		59 (74.4)	20 (25.3)	
Bachelor’s degree or higher	37 (17.6)	23 (62.2)	14 (37.8)		28 (75.7)	9 (24.3)	
Employment status				0.683			0.779
Unemployed	68 (32.4)	46 (67.6)	22 (32.4)		51 (75.0)	17 (25.0)	
Employed	142 (67.6)	92 (64.8)	50 (35.2)		109 (76.8)	33 (23.2)	
Monthly income				0.122			0.147
<10,000 baht	103 (49.0)	73 (70.9)	30 (29.1)		74 (71.8)	29 (28.2)	
≥10,000 baht (≈300 USD)	107 (51.0)	65 (60.7)	42 (39.3)		86 (80.4)	21 (19.6)	
Clinical factors							
Current smoking status				0.173			0.167
No	184 (87.6)	124 (67.4)	60 (32.6)		143 (77.7)	41 (22.3)	
Yes	26 (12.4)	14 (53.8)	12 (46.2)		17 (65.4)	9 (34.6)	
Current alcohol consumption				**0.008**			**0.012**
No	172 (81.9)	120 (69.8)	52 (30.2)		137 (79.7)	35 (20.3)	
Yes	38 (18.1)	18 (47.4)	20 (52.6)		23 (60.5)	15 (39.5)	
BMI				0.175			0.723
Normal (18.5–22.9 kg/m^2^)	88 (41.9)	51 (58.0)	37 (42.0)		64 (72.7)	24 (27.3)	
Underweight (<18.5 kg/m^2^)	65 (31.0)	45 (69.2)	20 (30.8)		52 (80.0)	13 (20.0)	
Overweight (23.0–24.9 kg/m^2^)	32 (15.2)	25 (78.1)	7 (21.9)		24 (75.0)	8 (25.0)	
Obese (≥25.0 kg/m^2^)	25 (11.9)	17 (68.0)	8 (32.0)		20 (80.0)	5 (20.0)	
Chronic diseases				0.496			0.939
No	104 (49.5)	66 (63.5)	38 (36.5)		79 (76.0)	25 (24.0)	
Yes	106 (50.5)	72 (67.9)	34 (32.1)		81 (76.4)	25 (23.6)	
Category of patient				0.774 **^†^**			0.494 **^†^**
New	187 (89.0)	122 (65.2)	65 (34.8)		144 (77.0)	43 (23.0)	
Relapse	13 (6.2)	10 (76.9)	3 (23.1)		8 (61.5)	5 (38.5)	
Multidrug-resistant (MDR) TB	10 (4.8)	6 (60.0)	4 (40.0)		8 (80.0)	2 (20.0)	
TB type				0.776 **^†^**			0.746 **^†^**
Pulmonary	196 (93.3)	128 (65.3)	68 (34.7)		150 (76.5)	46 (23.5)	
Extrapulmonary	14 (6.7)	10 (71.4)	4 (28.6)		10 (71.4)	4 (28.6)	
Duration of TB treatment				0.136			0.291
<1 month	42 (20.0)	27 (64.3)	15 (35.7)		29 (69.0)	13 (31.0)	
1–2 months	58 (27.6)	40 (69.0)	18 (31.0)		47 (81.0)	11 (19.0)	
3–6 months	82 (39.0)	48 (58.5)	34 (41.5)		60 (73.2)	22 (26.8)	
>6 months	28 (13.3)	23 (82.1)	5 (17.9)		24 (85.7)	4 (14.3)	
Psychosocial factors							
Social support from healthcare providers				**0.024**			**0.010**
Low to moderate level	97 (46.2)	56 (57.7)	41 (42.3)		66 (68.0)	31 (32.0)	
High level	113 (53.8)	82 (72.6)	31 (27.4)		94 (83.2)	19 (16.8)	
Social support from family				**0.010**			**0.020**
Low to moderate level	80 (38.1)	44 (55.0)	36 (45.0)		54 (67.5)	26 (32.5)	
High level	130 (61.9)	94 (72.3)	36 (27.7)		106 (81.5)	24 (18.5)	
Perceived stigma				**0.006**			0.465
None	74 (35.2)	60 (81.1)	14 (18.9)		60 (81.1)	14 (18.9)	
Low level	42 (20.0)	26 (61.9)	16 (38.1)		33 (78.6)	9 (21.4)	
Moderate level	69 (32.9)	38 (55.1)	31 (44.9)		50 (72.5)	19 (27.5)	
High level	25 (11.9)	14 (56.0)	11 (44.0)		17 (68.0)	8 (32.0)	

The bold parts indicate statistical significance at the 0.05 level. ^†^ Exact test.

**Table 3:** Association between independent variables and mental health problems in TB patients using logistic regression with forward Wald selection.

Factors	Univariable Analysis			Multivariable Analysis		
	Crude OR	95% CI	*p*-Value	Adjusted OR	95% CI	*p*-Value
Age						
<30 years	3.30	1.42, 7.68	0.006			
30–44 years	2.50	1.06, 5.98	0.039			
45–59 years	2.74	1.25, 6.00	0.012			
≥60 years	1		0.023			
Monthly income						
<10,000 baht	1					
≥10,000 baht	1.57	0.88, 2.80	0.123			
Current smoking status						
No	1					
Yes	1.77	0.77, 4.06	0.177			
Current alcohol consumption						
No	1					
Yes	2.56	1.25, 5.24	0.010			
BMI						
Normal (18.5–22.9 kg/m^2^)	1		0.183			
Underweight (<18.5 kg/m^2^)	0.61	0.31, 1.20	0.155			
Overweight (23.0–24.9 kg/m^2^)	0.39	0.15, 0.99	0.047			
Obese (≥25.0 kg/m^2^)	0.65	0.25, 1.66	0.367			
Duration of TB treatment						
<1 month	2.56	0.81, 8.11	0.111			
1–2 months	2.07	0.68, 6.32	0.201			
3–6 months	3.26	1.13, 9.43	0.029			
>6 months	1		0.152			
Social support from healthcare providers						
Low to moderate level	1					
High level	0.52	0.29, 0.92	0.025			
Social support from family						
Low to moderate level	1			1		
High level	0.47	0.26, 0.84	0.011	0.45	0.24, 0.83	0.010
Perceived stigma						
None	1		0.008	1		0.007
Low level	2.64	1.13, 6.18	0.026	2.77	1.16, 6.60	0.021
Moderate level	3.50	1.65, 7.41	0.001	3.56	1.66, 7.65	0.001
High level	3.37	1.26, 8.99	0.015	3.56	1.31, 9.67	0.013

**Table 4:** Association between independent variables and depression in TB patients using logistic regression with forward Wald selection.

Factors	Univariable Analysis			Multivariable Analysis		
	Crude OR	95% CI	*p*-Value	Adjusted OR	95% CI	*p*-Value
Monthly income						
<10,000 baht	1			1		
≥10,000 baht	0.62	0.33, 1.18	0.149	0.43	0.21, 0.87	0.020
Current smoking status						
No	1					
Yes	1.85	0.77, 4.45	0.172			
Current alcohol consumption						
No	1			1		
Yes	2.55	1.21, 5.40	0.014	2.90	1.27, 6.64	0.012
Social support from healthcare providers						
Low to moderate level	1			1		
High level	0.43	0.22, 0.83	0.011	0.44	0.22, 0.87	0.018
Social support from family						
Low to moderate level	1					
High level	0.47	0.25, 0.90	0.022			

## Data Availability

The data are available upon request from the corresponding author.
